# Comparison of bioelectrical body and visceral fat indices with anthropometric measures and optimal cutoffs in relation to hypertension by age and gender among Chinese adults

**DOI:** 10.1186/s12872-021-02100-8

**Published:** 2021-06-11

**Authors:** Binbin Zhang, Yaqi Fan, Yuxue Wang, Li Zhang, Chunjun Li, Jiangshan He, Pei Guo, Mianzhi Zhang, Minying Zhang

**Affiliations:** 1 School of Medicine, Nankai University, 94, Weijin Road, Tianjin, 300071 China; 2 Tianjin First Central Hospital, Tianjin, China; 3 Tianjin Union Medical Center, Tianjin, China; 4 Dongfang Hospital, Beijing University of Chinese Medicine, Beijing, China

**Keywords:** Body mass index, Waist-hip ratio, Percentage of body fat, Visceral fat area, Hypertension, Optimal cutoff value

## Abstract

**Background:**

Few studies have compared bioelectrical body and visceral fat indices with anthropometric measures, or evaluated their optimal cutoffs in relation to hypertension among Asians. We compared the efficiencies of bioelectrical indices (percentage of body fat, PBF; visceral fat area, VFA) with anthropometric measures (body mass index, BMI; waist-hip ratio, WHR) for hypertension and re-evaluated the optimal cutoffs of each index by age and gender.

**Methods:**

We conducted a cross-sectional survey among 8234 adults for health examination. PBF, VFA, BMI, WHR, and data on hypertension and behaviors were collected. Receiver operating characteristic (ROC) curve and areas under curves (AUCs) were used to analyze the efficiencies of the indices for hypertension, optimal cutoffs were estimated using the Youden index.

**Results:**

A total of 8234 individuals aged 21–91 with median age 44 (interquartile range [IQR] 33–56) years were included and 40.56% were men. The overall prevalence of hypertension was 27.47%. The studied indices were all associated with hypertension in all age-specific groups both among men and women except for WHR in 21–29 years old men and PBF in in 21–29 years old women. Among males, there were no statistical differences in powers of four indices for hypertension in all age-specific groups, except for 40–49 years, in which WHR was better than VFA. Among females, no differences were found among the indices in 30–39 and 70–79 years groups, while WHR was the best in 21–29 years group, VFA was better than PBF in 30–39 and 50–59 years groups, BMI was better than PBF and WHR in 60–69 years group. The optimal cutoffs of PBF, VFA, BMI and WHR ranged from 23.9 to 28.7%, 86.4 to 106.9cm^2^, 23.5 to 27.1 kg/m^2^, 0.92 to 0.96 across the age categories in males, and 32.8 to 36.3%, 75.9 to 130.9cm^2^, 21.9 to 26.4 kg/m^2^, 0.84 to 0.95 across the age categories in females, respectively.

**Conclusions:**

The obesity indices’ efficiencies for hypertension varied by age and gender, and their cutoff values varied across the age categories and gender. Specific indices and cutoffs based on person’s age and gender should be used to identify individuals with hypertension.

## Introduction

Hypertension, with increasingly high prevalence, has imposed a huge burden on the healthcare system globally [1]. Early identification and intervention have been proven to be feasible and cost-effective for hypertension control. Obesity has been demonstrated as a modifiable risk factor for hypertension [2–5]. Various indices are used to define obesity. BMI, recommended by the World Health Organization (WHO) [6], has been proven to be effective for distinguishing persons with hypertension and diabetes [7, 8]. However, BMI can only evaluate systemic obesity because it is not able to differentiate between muscle and fat mass. WHR is one of the markers of abdominal obesity and has demonstrated a stronger ability to identify cardiovascular and cerebrovascular diseases than BMI [9], indicating that it’s where the body fat accumulates rather than the body fat mass that relates to the above chronic diseases. PBF, which is the percentage of body fat weight relative to total body weight, has been proved more predictive for cardiovascular and cerebrovascular risks than BMI [10], but it cannot identify where the body fat stores. VFA measures the fat that stores around some important internal organs, including liver, intestine, and pancreas. Very few studies to date have examined the differences between VFA and the other indices on identifying hypertension.

The associations between each obesity index and hypertension are different due to their different perspectives measuring obesity. Therefore these obesity indices show different screening powers for hypertension [11–16]. Gender differences in associations between obesity indices and hypertension were found by some studies [11, 15, 16], though the evidences were equivocal [12–14]. Some studies found the efficiencies of obesity indices for identifying hypertension decreasing with age [12]. The associations between each obesity index and hypertension reported from different studies were inconsistent.

The optimal cutoffs of each obesity index for hypertension assessed by studies from different countries were diverse and varied by age and gender. For instance, the optimal cutoffs for BMI for hypertension were 24.1 and 24.9 kg/m^2^ for males, while 25.4 and 25.0 kg/m^2^ for females in Malaysia and west-north China, respectively [17, 18]. Similarly, the optimal cutoffs for PBF for hypertension were also found inconsistent, 30.0% and 40.0% were for Mexican men and women, 24.0% and 34.0% were for men and women from Inner Mongolia, China [19, 20]. Furthermore, the optimal cutoffs for VFA for hypertension were 134.6cm^2^ for men and 91.1cm^2^ for women in Korea [16], however, a study in China failed to identify an optimal cutoff of VFA for hypertension [21].

Very few studies have compared bioelectrical indices with anthropometric measures in relation to hypertension among Asians and the optimal cutoffs of obesity indices for hypertension by age and gender have rarely been studied. This study aims to compare the efficiency of bioelectrical indices (PBF, VFA) with the most commonly used anthropometric measures (BMI, WHR) in terms of identifying hypertension, and re-evaluated the optimal cutoff values of each obesity index by age and gender in Chinese adults.

## Methods

### Study design and population

The study included the baseline data of a cohort Study on natural Population in Beijing-Tianjin-Hebei Region, a National Key R&D Program of China. The study population were selected by cluster sampling from attendees at two health examination centers in Tianjin from September 2018 to December 2019. Individuals aged 18 years or elder who voluntarily participated in the survey were included in the study. Individuals were excluded if they (1) were with cognitive impairment, hearing impairment, articulate problems, or severe mental illness that cannot complete the survey; (2) were with heart pacemakers implanted; (3) could not stand independently. The research protocol was reviewed and approved by ethical committees from Nankai University and the hospitals in which the study was conducted, and written informed consent was got from each participant.

### Data collection

An investigator-administered questionnaire interview was conducted face-to-face to collect information including demographic characteristics (age, sex, ethnicity, marital status, highest education, occupation), self-reported personal and family history of hypertension (yes, no), alcohol and tobacco use history, and physical exercise (consistent, ≥ 3times/week; inconsistent, < 3times/week).

### Measurement

Height was measured to the nearest 0.1 cm without shoes using a calibrated stadiometer (GL-310, Seoul, Korea). Weight (0.1-kg precision) WHR, PBF, and VFA were measured by multifrequency bioelectric impedance method using the Inbody multifrequency impedance plethysmograph body composition analyzer (Inbody-770, Seoul, Korea). The participant stood barefoot on the foot electrode of the instrument in a fully vertical position with thin and light clothing, shared the weight evenly on both legs, held the hand electrode with both hands, and was prohibited from speaking during measurement. Measurement was completed after the reading was stable.

Blood pressure was measured in a sitting position for the right arm after resting for 5 to10 minutes, using blood pressure monitor (Kenz-AC OSC, Japan). Two readings were taken, 30 s apart, and a third measurement was conducted if the first two reads differed by more than 10 mmHg. The average of the two closest readings (1-mmHg precision) was used. Hypertension was defined using criteria from the 2010 Chinese guidelines for the management of hypertension [22]: systolic blood pressure (SBP) ≥ 140 mmHg and/or diastolic blood pressure (DBP) ≥ 90 mmHg or self-reported history of diagnosed hypertension.

### Statistical analyses

Statistical analyses were performed using the Statistical Package for Social Sciences (SPSS) version 24.0 (SPSS Inc., Chicago, IL, USA) and MedCalc (MedCalc Software, Mariakerke, Belgium). The normally distributed continuous variables were expressed as mean ± standard deviation (SD) and compared using t-test, while variables which were not normally distributed were described using median and quartile, and compared using rank-sum test. Categorical data were described as rate and proportion, and compared using Chi-square test. Logistic regression was used to assess the relationships between obesity indices and hypertension. The strength of the correlation was expressed as odds ratio (OR) and 95% confidence interval (CI). Before the analysis, the obesity indices were standardized (original data subtracted the average and then divided by the standard deviation), so the ORs indicated hypertension risk increased by per standard deviation.

ROC curve analysis was used to compare the predictive validity, and AUC was also measured to examine the screening power of each obesity index, and to describe the probability that an index would correctly identify subjects with hypertension. AUC was assessed with 0.5 as no power and 1.0 as perfect power. The optimal cut-off values were measured by the Youden index, which was calculated with the sensitivity and specificity of the indices at various cut-off points. The method suggested by DeLong et al. was used to test whether the differences between AUC values were statistically significant [23]. Two-tailed *P*-values ≤ 0.05 were considered statistically significant.

## Results

**Fig. 1:**
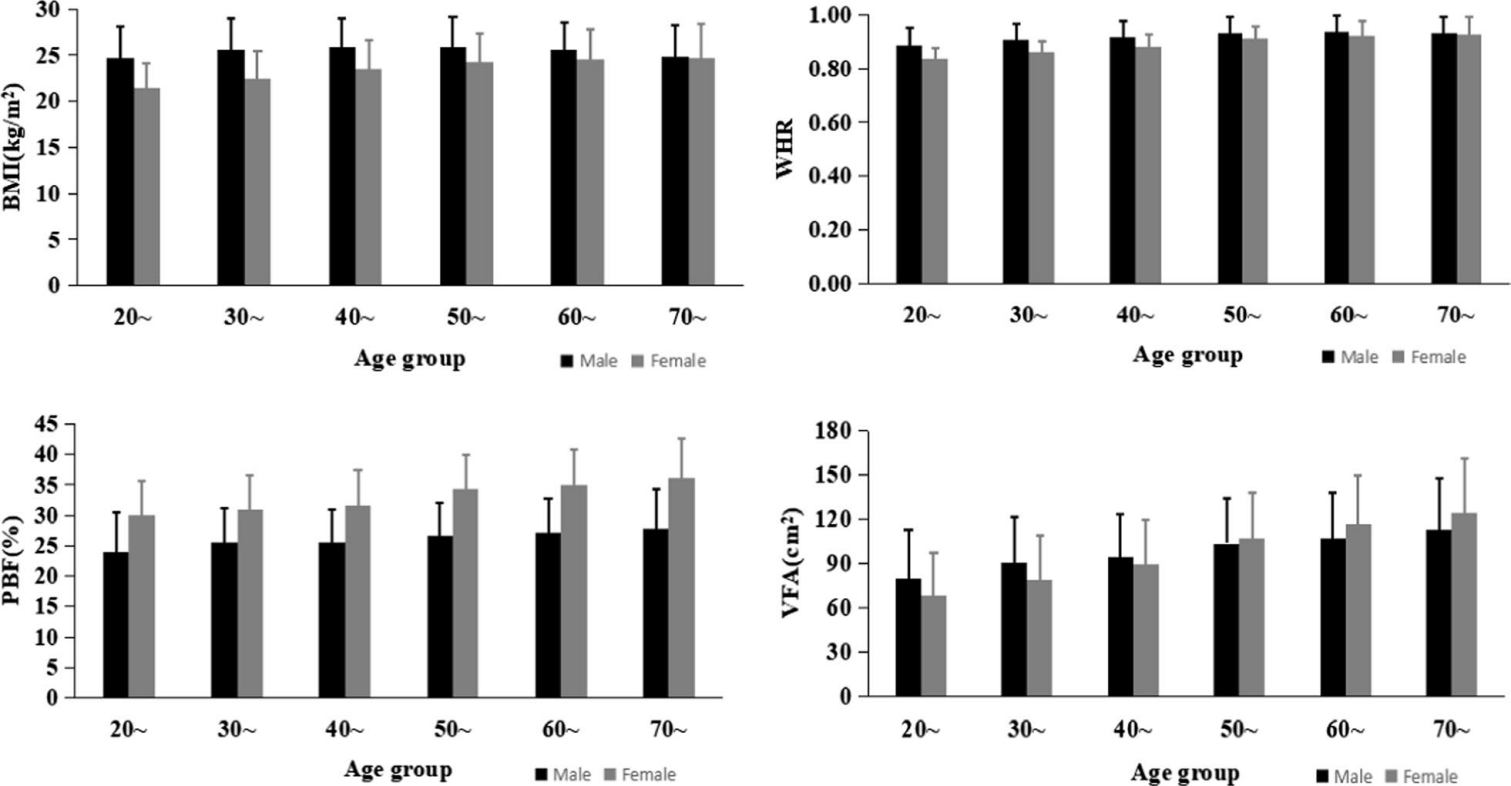
The age and gender stratified mean PBF, VFA, BMI and WHR

As summarized in Table 1, 8234 individuals aged 21–91 years with median age 44 (IQR 33–56) years, were included and 40.56% were men. The overall prevalence of hypertension was 27.47% and increased with age and decreased with education level. The prevalence of hypertension was higher in men (35.50%) than in women (21.31%). In a current marriage, smoking, alcohol drinking, and family history of hypertension were all positively associated with hypertension. The mean PBF, VFA, BMI and WHR were 29.71 ± 6.71%, 93.21 ± 34.35cm^2^, 24.12 ± 3.45 kg/m^2^ and 0.89 ± 0.06 respectively. The age and gender stratified mean PBF, VFA, BMI and WHR were presented in Fig. 1. Compared with women, men were characterized by a higher prevalence of hypertension, smoking, alcohol drinking, regular exercise and having higher BMI and WHR, but lower PBF (all *P *values < 0.01). No statistical difference of VFA was found between different genders.

**Table 1:** Hypertension prevalence by characteristics of the participants

Items	Total (N = 8234)		Men (n = 3340)		Women (n = 4894)	
	N (%)	Hypertension (prevalence, %)	N (%)	Hypertension (prevalence, %)	N (%)	Hypertension (prevalence, %)
Age, (year)						
21–29	1182 (14.36)	104 (8.80) ^+^	423 (12.66)	68 (16.08)^+^	759 (15.51)	36 (4.74) *^+^
30–39	2418 (29.37)	306 (12.66)	949 (28.41)	199 (20.97)	1469 (30.02)	107 (7.28)
40–49	1600 (19.43)	357 (22.31)	625 (18.71)	203 (32.48)	975 (19.92)	154 (15.79)
50–59	1430 (17.37)	492 (34.41)	555 (16.62)	234 (42.16)	875 (17.88)	258 (29.49)
60–69	1034 (12.56)	582 (56.29)	469 (14.04)	283 (60.34)	565 (11.54)	299 (52.92) *
70–91	570 (6.92)	421 (73.86)	319 (9.55)	232 (72.73)	251 (5.13)	189 (75.30) *
Educational level						
Middle school or below	734 (8.91)	386 (52.59) ^+^	331 (9.91)	186 (56.19) ^+^	403 (8.23)	200 (49.63) *^+^
College or undergraduate	4915 (59.69)	1431 (29.11)	1929 (57.75)	759 (39.35)	2986 (61.01)	672 (22.51) *
Postgraduate or above	2585 (31.39)	445 (17.21)	1080 (32.34)	274 (25.37)	1505 (30.75)	171 (11.36)
Han Ethnicity	7938 (96.41)	2193 (27.63)	3233 (96.80)	1190 (36.81)^+^	4705 (96.14)	1003 (21.32)
Occupation						
Civil servant	2066 (25.09)	423 (20.47) ^+^	905 (27.10)	270 (29.83) ^+^	1161 (23.72)	153 (13.18) *^+^
Professionals	3775 (45.85)	667 (17.67)	1399 (41.89)	374 (26.73)	2376 (48.55)	293 (12.33) *
Retired staff	1702 (20.67)	1006 (59.11)	749 (22.43)	480 (64.09)	953 (19.47)	526 (55.19) *
Others	691 (8.39)	166 (24.02)	287 (8.59)	95 (33.10)	404 (8.26)	71 (17.57)
In a current marriage	7001 (85.03)	2066 (29.51)^+^	2916 (87.31)	1121 (38.44)^+^	4085 (83.47)	945 (23.13) *^+^
Smoking	909 (11.04)	343 (37.73)^+^	751 (22.49)	293 (39.01)	158 (3.23)	50 (31.65) *^+^
Alcohol drinking	1324 (16.08)	455 (34.37)^+^	946 (28.32)	370 (39.11)^+^	378 (7.72)	85 (22.49) *
Regular Exercise	2327 (28.26)	787 (33.82)^+^	1171 (35.06)	463 (39.54)^+^	1156 (23.62)	324 (28.03) *^+^
Family history of hypertension	3890 (47.24)	1394 (35.84)^+^	1516 (45.39)	711 (46.90)^+^	2374 (48.51)	683 (28.77) *^+^
Obesity index ($\bar{x}\pm SD)$						
PBF (%)	29.71 ± 6.71		25.99 ± 5.83		32.25 ± 6.05*	
VFA (cm^2^)	93.21 ± 34.35		96.35 ± 32.87		91.07 ± 35.17	
WHR	0.89 ± 0.06		0.92 ± 0.06		0.88 ± 0.06*	
BMI (kg/m^2^)	24.12 ± 3.45		25.52 ± 3.25		23.16 ± 3.25*	

Data were presented as mean ± SD or n (%)

*PBF* percentage body fat, *VFA* visceral fat area, *BMI* body mass index, *WHR* waist-hip ratio

^+^*P* < 0.05 for comparison of the prevalence of hypertension among different categories of the specific characteristics

*P < 0.05 for comparison of the prevalence of hypertension between men and women of the specific characteristics

Logistic regression was used to analyze the relationship between obesity indices and hypertension by gender. Both standardized crude ORs and ORs adjusted by age, marital status, ethnicity, education level, occupation, smoking, alcohol drinking, physical exercise, and family history of hypertension were calculated. For each obesity index, the crude and adjusted ORs for hypertension were significantly higher than the reference level of 1.00 in both men and women, a 1-SD increase of each index was associated with increased risk of hypertension in both genders.

Comparisons of ORs for all obesity indices based on Z-score standardization by gender were also conducted. As shown in Table 2, no significant differences of crude and adjusted ORs were found among all obesity indices both in men and women, and no significant difference of crude and adjusted ORs was found for each obesity index between different genders.

**Table 2:** Standardized odds ratios and 95% confidence intervals of obesity indices for hypertension by gender

	Men	Women
PBF z-score		
Crude OR	1.76 (1.63–1.90)*	1.94 (1.79–2.09)*
Adjusted OR	1.62 (1.48–1.76)*	1.42 (1.30–1.55)*
VFA z-score		
Crude OR	1.89 (1.75–2.05)*	2.29 (2.12–2.47)*
Adjusted OR	1.56 (1.44–1.70)*	1.55 (1.42–1.69)*
BMI z-score		
Crude OR	1.55 (1.43–1.67)*	1.92 (1.79–2.06)*
Adjusted OR	1.63 (1.49–1.77)*	1.54 (1.42–1.67)*
WHR z-score		
Crude OR	1.91 (1.76–2.06)*	2.36 (2.18–2.55)*
Adjusted OR	1.63 (1.50–1.78)*	1.48 (1.35–1.62)*

Adjusted for age, marital status, ethnicity, education, occupation, smoking, alcohol drinking, physical exercise and family history of hypertension

*PBF* percentage body fat, *VFA* visceral fat area, *BMI* body mass index, *WHR* waist-hip ratio

Symbols denote significant of ORs (^⁎^*P* < 0.0001)

Table 3 showed the optimal cut-off values, corresponding AUCs, sensitivities, specificities, and Youden indexes of each obesity index for identifying hypertension by gender. WHR and VFA presented greater AUCs than BMI and PBF (*P* < 0.001) both in men and women, and there was no difference between the identifying power of WHR and VFA in males and females. However, among men, PBF had larger AUC than BMI, whereas they showed no difference among women.

**Table 3:** Receiver operating characteristic curve analysis of the obesity indices for identifying subjects with hypertension by gender

	AUC (95%CI)	*P*	Optimal cut-off point	Sensitivity (%)	Specificity (%)	Youden index
Men						
PBF (%)	0.65 (0.64–0.67)	< 0.001	27.0	56.93	66.10	0.23
VFA (cm^2^)	0.67 (0.66–0.69)	< 0.001	96.1	63.90	63.18	0.27
BMI (kg/m^2^)	0.62 (0.60–0.64)	< 0.001	25.1	63.25	54.31	0.18
WHR	0.68 (0.66–0.70)	< 0.001	0.91	66.94	61.53	0.28
Women						
PBF (%)	0.68 (0.66–0.70)	< 0.001	33.8	62.51	65.31	0.28
VFA (cm^2^)	0.73 (0.71–0.75)	< 0.001	91.7	71.81	62.43	0.34
BMI (kg/m^2^)	0.69 (0.67–0.71)	< 0.001	23.3	66.25	63.15	0.29
WHR	0.72 (0.71–0.74)	< 0.001	0.89	62.99	69.36	0.32

*PBF* percentage body fat, *VFA* visceral fat area, *BMI* body mass index, *WHR* waist-hip ratio

Table 4 showed the standardized ORs and 95% CIs of the four indices for hypertension by age and gender. The adjusted ORs were calculated with control of potential confounding variables, including age, ethnicity, marital status, education level, occupation, smoking, alcohol drinking, physical exercise and family history of hypertension. Among men, with the exception of adjusted WHR in 21–29 years group, the other indices were significantly correlated with hypertension risk. In women, significant differences were observed between all obesity indices and hypertension, except for PBF in 21–29 years group.

**Table 4:** Standardized odds ratios and 95% confidence intervals of obesity indices for hypertension by age and gender

	Age group (years)					
	21–29	30–39	40–49	50–59	60–69	70–91
Men						
PBF z-score						
Crude OR	1.47** (1.13–1.91)	1.73*** (1.47–2.05)	1.86*** (1.54–2.25)	1.43*** (1.19–1.70)	1.82*** (1.48–2.25)	1.41** (1.09–1.81)
Adjusted OR	1.47** (1.10–1.94)	1.76*** (1.48–2.10)	1.83*** (1.51–2.23)	1.37** (1.14–1.65)	1.11** (1.03–1.20)	1.43* (1.09–1.88)
VFA z-score						
Crude OR	1.35* (1.06–1.73)	1.74*** (1.49–2.03)	1.83*** (1.52–2.20)	1.45*** (1.21–1.73)	1.62*** (1.33–1.98)	1.38* (1.07–1.78)
Adjusted OR	1.32* (1.01–1.71)	1.75*** (1.48–2.06)	1.73*** (1.43–2.10)	1.37*** (1.14–1.66)	1.55*** (1.25–1.93)	1.37* (1.04–1.81)
BMI z-score						
Crude OR	1.45** (1.13–1.86)	1.69*** (1.45–1.98)	1.99*** (1.63–2.42)	1.48*** (1.23–1.78)	1.91*** (1.54–2.38)	1.49** (1.14–1.95)
Adjusted OR	1.40* (1.07–1.84)	1.67*** (1.42–1.96)	1.94*** (1.58–2.38)	1.44*** (1.19–1.74)	1.92*** (1.52–2.44)	1.45* (1.08–1.94)
WHR z-core						
Crude OR	1.34* (1.05–1.71)	1.76*** (1.50–2.06)	2.01*** (1.67–2.43)	1.53*** (1.28–1.84)	1.76*** (1.43–2.17)	1.50** (1.16–1.93)
Adjusted OR	1.29 (0.99–1.68)	1.72*** (1.46–2.03)	1.89*** (1.56–2.30)	1.47*** (1.21–1.79)	1.77*** (1.41–2.22)	1.53** (1.16–2.03)
Women						
PBF z-score						
Crude OR	1.20 (0.86–1.68)	1.34** (1.10–1.63)	1.77*** (1.47–2.13)	1.29** (1.11–1.51)	1.39*** (1.17–1.65)	1.45* (1.08–1.94)
Adjusted OR	1.16 (0.81–1.65)	1.35** (1.09–1.67)	1.73*** (1.42–2.10)	1.29** (1.10–1.52)	1.16** (1.08–1.25)	1.49* (1.09–2.03)
VFA z-score						
Crude OR	1.46** (1.10–1.93)	1.45*** (1.22–1.72)	1.64*** (1.40–1.93)	1.43*** (1.24–1.66)	1.52*** (1.27–1.82)	1.43* (1.06–1.94)
Adjusted OR	1.39* (1.01–1.90)	1.44*** (1.20–1.74)	1.64*** (1.38–1.94)	1.45*** (1.24–1.69)	1.47*** (1.22–1.77)	1.48* (1.07–2.06)
BMI z-score						
Crude OR	1.44** (1.09–1.89)	1.36** (1.14–1.62)	1.73*** (1.47–2.04)	1.47*** (1.26–1.71)	1.72*** (1.43–2.07)	1.60** (1.17–2.20)
Adjusted OR	1.39* (1.04–1.88)	1.36** (1.12–1.65)	1.73*** (1.46–2.06)	1.46*** (1.25–1.71)	1.68*** (1.38–2.05)	1.68** (1.21–2.34)
WHR z-core						
Crude OR	1.55** (1.16–2.06)	1.43*** (1.18–1.72)	1.84*** (1.53–2.22)	1.33** (1.15–1.55)	1.35** (1.14–1.60)	1.38* (1.02–1.85)
Adjusted OR	1.45* (1.04–2.02)	1.37** (1.12–1.68)	1.78*** (1.46–2.17)	1.33** (1.14–1.56)	1.37** (1.13–1.65)	1.49* (1.07–2.07)

Adjusted for age, marital status, ethnicity, education, occupation, smoking, alcohol drinking, physical exercise and family history of hypertension

*PBF* percentage body fat, *VFA* visceral fat area, *BMI* body mass index, *WHR* waist-hip ratio

Symbols denote significant of ORs (^⁎^*P* < 0.05; ^⁎⁎^*P* < 0.01; ^⁎⁎⁎^*P* < 0.0001)

Table 5 showed the optimal cut-off points of each obesity index for hypertension risk, corresponding AUCs, sensitivities, specificities and the Youden indexes by gender and age-specific group. Among males, there were no statistical differences in the screening powers of four indices for hypertension in all age-specific groups, except for 40–49 years, in which WHR was better than VFA (*P* < 0.01)*.* Among females, no differences were found among the indices in 40–49 and 70–91 years groups, while WHR was the best in 21–29 years group (all *P* < 0.01), VFA was better than PBF in 30–39 and 50–59 years groups (all *P* < 0.01), BMI was better than PBF and WHR in 60–69 years group (all *P* < 0.01). In addition, the optimal cutoffs of each index varied by gender and age. In males, the optimal cutoffs of PBF ranged from 23.9 to 28.7% with the largest in 70–91 years group and the smallest in the 21–29 years group; the optimal cutoffs of VFA ranged from 86.4 to 106.9cm^2^, with the largest in the 60–69 years group and the smallest in the 50–59 years group; the optimal cutoffs of BMI ranged from 23.5 to 27.1 kg/m^2^, with the largest in 40–49 years group and the smallest in 21–29 years group; the optimal cutoffs of WHR ranged from 0.92 to 0.96, with the largest in 60–69 years group and the smallest in 30–39 and 40–49 years groups. In females, the optimal cutoffs of PBF ranged from 32.8 to 36.3% with the largest in 50–59 years group and the smallest in the 40–49 years group; the optimal cutoffs of VFA ranged from 75.9 to 130.9cm^2^, with the largest in the 70–91 years group and the smallest in the 21–29 years group; the optimal cutoffs of BMI ranged from 21.9 to 26.4 kg/m^2^, with the largest in 50–59 years group and the smallest in 21–29 years group; the optimal cutoffs of WHR ranged from 0.84 to 0.95, with the largest in 60–69 years group and the smallest in 21–29 and 30–39 years groups.

**Table 5:** Receiver operating characteristic curve analysis of the obesity indices for hypertension by age and gender

	AUC (95%CI)	P	Optimal cutoff point	Sensitivity (%)	Specificity (%)	Youden index
Men						
21–29 years						
PBF (%)	0.63 (0.56–0.70)	0.001	23.9	70.59	54.37	0.25
VFA (cm^2^)	0.61 (0.54–0.68)	0.003	86.4	52.94	66.48	0.19
BMI (kg/m^2^)	0.62 (0.55–0.69)	0.001	23.5	80.88	41.69	0.23
30–39 years						
PBF (%)	0.65 (0.61–0.69)	< 0.001	25.2	68.84	52.53	0.21
VFA (cm^2^)	0.66 (0.61–0.70)	< 0.001	88.4	66.33	57.47	0.24
BMI (kg/m^2^)	0.64 (0.60–0.68)	< 0.001	26.8	47.74	73.60	0.21
WHR	0.66 (0.62–0.70)	< 0.001	0.92	52.76	73.07	0.26
40–49 years						
PBF (%)	0.67 (0.62–0.71)	< 0.001	25.0	71.92	54.50	0.26
VFA (cm^2^)	0.66 (0.61–0.70)	< 0.001	96.1	61.08	64.45	0.26
BMI (kg/m^2^)	0.67 (0.62–0.71)	< 0.001	27.1	47.78	76.07	0.24
WHR	0.70 (0.65–0.74)	< 0.001	0.92	60.59	71.80	0.32
50–59 years						
PBF (%)	0.61 (0.56–0.66)	< 0.001	27.2	57.69	62.31	0.20
VFA (cm^2^)	0.60 (0.56–0.65)	< 0.001	87.5	80.77	38.32	0.19
BMI (kg/m^2^)	0.62 (0.57–0.67)	< 0.001	25.8	59.83	59.81	0.20
WHR	0.62 (0.58–0.67)	< 0.001	0.95	37.61	81.93	0.20
60–69 years						
PBF (%)	0.65 (0.60–0.70)	< 0.001	26.6	61.13	61.29	0.22
VFA (cm^2^)	0.620 (0.57–0.67)	< 0.001	106.9	58.30	59.68	0.18
BMI (kg/m^2^)	0.65 (0.61–0.70)	< 0.001	25.6	56.54	65.05	0.22
WHR	0.65 (0.60–0.70)	< 0.001	0.96	35.34	86.56	0.22
70–91 years						
PBF (%)	0.60 (0.53–0.67)	0.005	28.7	51.72	65.52	0.17
VFA (cm^2^)	0.59 (0.52–0.66)	0.012	85.9	80.60	36.78	0.17
BMI (kg/m^2^)	0.63 (0.56–0.70)	0.001	25.0	53.88	66.67	0.21
WHR	0.61 (0.54–0.68)	0.002	0.93	55.17	65.52	0.21
Women						
21–29 years						
VFA (cm^2^)	0.61 (0.52–0.71)	0.022	75.9	50.00	71.65	0.22
BMI (kg/m^2^)	0.58 (0.47–0.69)	0.116	21.9	52.78	66.39	0.19
WHR	0.66 (0.58–0.74)	0.001	0.84	61.11	67.50	0.29
30–39 years						
PBF (%)	0.58 (0.52–0.64)	0.006	33.4	49.53	68.58	0.18
VFA (cm^2^)	0.60 (0.54–0.66)	< 0.001	88.0	53.27	69.60	0.23
BMI (kg/m^2^)	0.58 (0.51–0.64)	0.009	24.3	42.06	78.05	0.20
WHR	0.60 (0.54–0.65)	0.001	0.84	72.90	41.85	0.15
40–49 years						
PBF (%)	0.66 (0.62–0.71)	< 0.001	32.8	64.94	62.36	0.27
VFA (cm^2^)	0.67 (0.63–0.72)	< 0.001	82.8	78.57	52.25	0.31
BMI (kg/m^2^)	0.69 (0.64–0.73)	< 0.001	23.3	72.73	59.93	0.33
WHR	0.67 (0.63–0.72)	< 0.001	0.87	78.57	48.72	0.27
50–59 years						
PBF (%)	0.57 (0.53–0.61)	0.001	36.3	44.96	66.61	0.12
VFA (cm^2^)	0.59 (0.55–0.63)	< 0.001	122.9	37.98	77.80	0.16
BMI (kg/m^2^)	0.59 (0.55–0.63)	< 0.001	26.4	29.46	85.58	0.15
WHR	0.58 (0.53–0.62)	< 0.001	0.92	44.96	67.91	0.13
60–69 years						
PBF (%)	0.60 (0.55–0.64)	< 0.001	35.6	55.52	62.03	0.18
VFA (cm^2^)	0.62 (0.57–0.66)	< 0.001	126.6	44.48	77.82	0.22
BMI (kg/m^2^)	0.64 (0.60–0.69)	< 0.001	25.2	46.82	75.19	0.22
WHR	0.59 (0.54–0.63)	< 0.001	0.95	32.44	82.33	0.15
70–91 years						
PBF (%)	0.62 (0.54–0.70)	0.004	36.1	55.56	69.35	0.25
VFA (cm^2^)	0.60 (0.53–0.68)	0.015	130.9	47.62	74.19	0.22
BMI (kg/m^2^)	0.64 (0.56–0.72)	0.001	21.8	84.13	40.32	0.24
WHR	0.58 (0.50–0.67)	0.046	0.89	70.37	48.39	0.19

*PBF* percentage body fat, *VFA* visceral fat area, *BMI* body mass index, *WHR* waist-hip ratio

As shown in Table 5, the AUCs of all obesity indices tested statistically significant in the logistic regression models were > 0.5 and statistically significant in all age-specific groups except for BMI in 21–29 years old women. Among men, in each age-specific groups, the obesity indices had no statistically significant differences in identifying subjects with hypertension, except for 40–49 group, in which WHR was the best. Among women, WHR had the best efficiency for hypertension in 21–29 years group, followed by VFA, while BMI was not statistically significant; in 30–39 years and 50–59 years groups, VFA was significantly more powerful than PBF; in 60–69 age group, the discrimination power of BMI was higher than both PBF and WHR.

## Discussion

The current study found that though the studied obesity indices were all associated with hypertension, their efficiencies to distinguish persons of hypertension and the optimal cutoffs varied by age and gender. The results revealed that we should use different indices and cutoff values for persons with different characteristics to identify individuals with hypertension more accurately. Furthermore, it suggested that people who try to prevent hypertension by controlling their weight should choose the best indices and cutoff values based on their specific age and gender.

Studies have proven that obesity indices are strongly associated with hypertension and can be used to identify the disease [12, 24–29]. To date, the results on which index is the best for identifying hypertension are inconsistent. Dutra Maurílio et al. [25] suggested that abdominal fat was more strongly associated with hypertension than BMI in both men and women while Zhou et al. [26] found that waist-to-height ratio (WHtR) had the best efficiency in identifying hypertension in men while BMI was the best in women. These differences might be related to race, but the impact of age could not be ignored. The current study found that there were differences not only in gender but also in age regarding the ability of obesity indexes to identify hypertension. Moreover, the abilities of obesity indices to identify hypertension varied more in women than in men. The gender differences had already been proven by previous studies [24, 26, 27], but very few studies examined the age-specific differences among various obesity indices [25–29]. Age was not only a key risk factor for hypertension but also strongly related to obesity. Both gender and age have impact on the amount and how human body accumulates fat. Therefore, bias might occur if the influence of age was not taken into account when studying the relationship between obesity and hypertension. In addition, we found the optimal cutoff values of the studied indices for hypertension varied by not only gender but also age, the optimal cutoff values in different age-specific groups of the same gender were various. So, it is not advisable to directly judge the cutoff values without age grouping and reassessment of cutoff values for hypertension with age-grouping in other races is necessary. Our study suggested that it is crucial to use the appropriate indices and optimal cutoffs based on the individual’s specific age and gender for hypertension screening and prevention.

Dong et al. found that the AUCs of obesity indices decreased with age among both men and women [24]. Wu et al. suggested that obesity index had a stronger ability to identify hypertension in young people than in middle-aged people [12]. But we found that though the association between obesity indices and hypertension varied by age, there was no linear trend in it. Possible reasons for this result might be related to the complex etiology of hypertension, besides obesity, genetic factors, deterioration of vital organs, changes in vascular structure and function, changes in intestinal flora, and increased salt sensitivity also play important roles in the development of hypertension [30–33]. The biological mechanisms underlying the age differences of obesity indices in distinguishing persons with hypertension remain to be explored.

We found that among the four indices, VFA had the smallest AUCs in men but relative larger AUCs in women elder than 40 years, indicating that VFA was more powerful in identifying hypertension among women than among men. We speculated that the gender difference might be related to the sexually different structure of the human body. Among Asians, compared with men, women tend to store more fat in the subcutaneous tissues around the waist, hips, and thighs [34], so excess visceral fat may be more predictive of chronic disease in women than in men. In addition, we found that the VFA's advantage in identifying hypertension in women was gradually replaced by BMI with age. The reason for this change might be related to the secretion of free fatty acids and angiotensinogen, and sympathetic nervous system activation [35], which could slowly decrease the influence of visceral fat. Given the superiority of VFA in identifying females with hypertension, the mechanism of visceral fat's influence on female hypertension is worthy of further study.

As an un-invasive method, BIA works well in healthy subjects and in patients with stable water [36]. To control the measurement errors caused by fluid instability of hypertension, we asked each participant if they had chronic kidney disease (CKD) and no participant reported having CKD. In addition, we estimated glomerular filtration rate and found it was > 90 ml/min/1.73m^2^ for each participant, indicating all participants had normal kidney function. Third, participants were instructed to fast for ≥ 12 h and no strenuous activity before measurement the following morning. The measurement data showed that all participants has normal Extracellular Water (ECW). Though measurement errors caused by fluid instability of the study subjects could not be completely eliminated when performing BIA measurements, changes of body water caused by CKD, exercise, sweating, and drinking were excluded in the study [37].

To our knowledge, the current study was the first to compare BMI, WHR, PBF and VFA in identifying hypertension by gender and age in Asian adults of a broader range of age-specific categories, which helped to find out the age and gender-specific best index. In addition, it was the first to assess the age and gender-specific optimal cutoff values of BMI, WHR, PBF and VFA for hypertension. However, this study also had some limitations. First, designed as a cross-sectional study, it was impossible to evaluate the temporal sequence and causal relationship between obesity and hypertension. The results needed to be further proved by cohort study. Second, due to the lack of relevant information, this study had not adjusted confounding factors including dietary pattern [38] or intake of sodium [39] and potassium [40]. Third, Using BIA as the principle to estimate body fat, the estimation error could not be eliminated and it would vary with the change of body fat level. But compared with Computerized tomography (CT) and Magnetic resonance imaging (MRI), BIA is relatively inexpensive, free of radiation, has very limited between observer variations and can be performed easily. Therefore it may be the most applicable method for large-scale studies [41]. Despite the limitations, the findings had public health relevance and may be valuable in developing more accurate and specific public health recommendations and preventative interventions.

## Conclusions

Although PBF, VFA, BMI, and WHR were all positively correlated with hypertension, their power of identifying hypertension and the optimal cutoffs varied by age and gender. It should highlight to use age and gender specific indices and cutoff values to distinguish persons with hypertension.

## Data Availability

The datasets used and/or analysed during the current study are available from the corresponding author on reasonable request.

## Abbreviations

PBF: Percentage of body fat
VFA: Visceral fat area
BMI: Body mass index
WHR: Waist-hip ratio
WHtR: Waist-to-height ratio
ROC: Receiver operating characteristic
AUCs: Areas under curves
WHO: World Health Organization
SBP: Systolic blood pressure
DBP: Diastolic blood pressure
SPSS: Statistical Package for Social Sciences
SD: Standard deviation
OR: Odds ratio
CI: Confidence interval
